# Curcumin-Based Tri-Allyl Derivative for Bismaleimide Resins: Synergistic Modulation of Thermal Stability and Fire Safety

**DOI:** 10.3390/polym18030399

**Published:** 2026-02-03

**Authors:** Hui Liu, Teresa Olszewska, Hao Liu

**Affiliations:** 1 State Key Laboratory of Advanced Fibers Materials, Center for Advanced Low-Dimension Materials, College of Materials Science and Engineering, Donghua University, Shanghai 201620, China; 2 Department of Organic Chemistry, Faculty of Chemistry, Gdańsk University of Technology, 80-233 Gdańsk, Poland

**Keywords:** bismaleimide resin, curcumin, bio-based polymer, flame retardancy, char yield

## Abstract

Bio-based bismaleimide (BMI) resins can reduce environmental impact and impart intrinsic flame retardancy, but achieving a high glass transition temperature (*T*g) remains challenging. Here, we replace the conventional petrochemical co-monomer O,O′-diallyl bisphenol A (DABPA) with a synthesized tri-allyl derivative of curcumin (AEC) in 4,4′-bismaleimidodiphenylmethane (BDM)-based resins. The AEC monomer, synthesized via exhaustive O- and C-alkylation of curcumin, acts as a trifunctional crosslinker. By systematically varying the imide:allyl molar ratio, we optimized the network properties. We optimize the network’s thermal and fire-safety properties. The optimized formulation (BDM: AEC = 1:0.87, denoted BA-0.87) yields 43.06% char at 800 °C and reduces the peak heat release rate (PHRR) by 13.2% compared to the conventional BDM/DABPA control (BD-0.87). Meanwhile, BA-0.87 passes UL-94 V-0 with no dripping and attains a *T*g above 400 °C—nearly 100 °C higher than BD-0.87. These enhancements arise from curcumin’s rigid conjugated structure, which increases crosslink density and promotes char formation during decomposition. Our work demonstrates a viable, bio-derived pathway to engineer BMI resins that simultaneously improve thermal stability and intrinsic flame retardancy. Such resins are promising for demanding aerospace and high-temperature electronic applications that require both fire safety and stability.

## 1. Introduction

Bismaleimide (BMI) resins are indispensable matrices for high-temperature composites, printed circuit boards, and structural adhesives due to their exceptional thermal stability beyond 300 °C [1,2,3,4]. However, BMI networks are inherently brittle, which limits their broader adoption. Meanwhile, safety regulations in aerospace and electronics (e.g., IEC flame-retardancy standards) demand superior flame resistance [5]. Thus, there is a pressing need for BMI formulations that combine mechanical hardness and intrinsic flame retardancy without compromising other properties.

Currently, the industry-standard toughening co-monomer is O,O′-diallyl bisphenol A (DABPA), which copolymerizes with maleimide via allyl–maleimide reactions [6]. However, DABPA is derived from bisphenol A (BPA), a compound known for endocrine-disruption and reproductive toxicity [7,8]. Regulatory frameworks like EU REACH have severely restricted BPA use, making the development of BPA/DABPA-free BMI systems both necessary and urgent [9]. Efforts to find bio-based BMI modifiers have shown promise. For example, a propargyl ether derived from the natural phenol honokiol copolymerized with BMI to yield networks with *T*g > 440 °C and storage modulus > 1.3 GPa at 400 °C, highlighting the benefit of rigid phenolics [10]. Similarly, a resveratrol-based allyl ether gave a BMI network with *T*g ≈ 388 °C, char yield ≈ 48.6%, UL-94 V-0 rating, and limiting oxygen index (LOI) of 37.4%, demonstrating excellent flame retardancy [11]. Other biocompatible modifiers (e.g., from cardanol or castor oil) have also been explored to balance crosslink density and storage modulus [12,13].

Despite these advances, no single biomolecule has yet been shown to enhance thermal stability and intrinsic flame retardancy simultaneously through rational network design. Comparative studies consistently report that gains in one performance dimension (e.g., higher LOI or UL-94 class) often incur losses in another (e.g., reduced toughness) [11,14,15,16].

Curcumin, a natural polyphenol from *Curcuma longa*, is a compelling candidate to bridge this gap [17,18,19]. It contains two phenolic –OH groups, activated by methoxy substituents, which can undergo Michael addition with maleimides. Its rigid conjugated backbone, including enol and β-diketone forms, promotes crosslink density and char formation during decomposition [20,21]. Unlike bisphenols, curcumin possesses a favorable biocompatibility profile. Recent comprehensive reviews, such as Neganova et al. (2025), have highlight its low toxicity, anti-tumor, and antioxidant activities, positioning it as a safe precursor for next-generation materials [22]. While curcumin has been used to impart anticorrosion, antifouling, and biocompatible properties in epoxies, benzoxazines, and polyurethanes, its potential to simultaneously optimize processing, thermal, and flame-retardant properties in BMI networks has not been explored [23,24,25].

**Figure 1:**
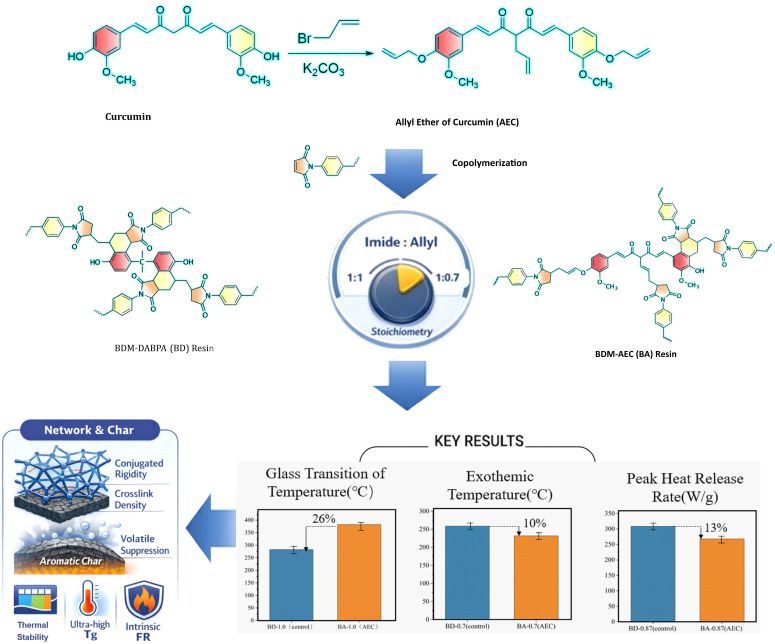
Schematic representation of the BA and BD resins.

In this study, we synthesized a tri-allyl derivative of curcumin (AEC) via exhaustive O- and C-alkylation, which acts as a trifunctional crosslinker, and copolymerized it with 4,4′-bismaleimidodiphenylmethane (BDM) at varying imide:allyl molar ratios (0.7, 0.87, 1.0), as shown in Figure 1. We demonstrate that at an imide:allyl ratio of 0.87, the AEC-modified BMI (BA-0.87) achieves outstanding performance, *T*_g_ > 400 °C, LOI ≈ 37.4%, and UL-94 V-0 classification, while maintaining an exothermic temperature as lower than the conventional BDM/DABPA reference (BD-0.87). In other words, introducing AEC eliminates the traditional trade-off between thermal resistance and flame retardancy. Comparative analysis against BDM/DABPA benchmarks shows that curcumin’s intrinsic char-forming capacity and rigid structure confer superior thermo-oxidative stability without sacrificing manufacturability. These findings advance the rational design of bio-based thermosetting networks by revealing how phenolic allyl ether stoichiometry controls crosslink architecture and provides a pathway to phase out endocrine-disrupting comonomers in safety-critical composites for applications (e.g., aerospace prepregs, high-frequency laminates, fire-resistant adhesives) subjected to sustained temperatures above 300 °C.

## 2. Experimental

### 2.1. Raw Materials

4,4′-bismaleimidodiphenylmethane (BDM, 98% purity) and O,O′-diallyl bisphenol A (DABPA, 90% purity) was supplied by Joson (Shanghai) Electronics Technology Co., Ltd. (Shanghai, China). Curcumin (98% purity) was purchased from Bide Pharmatech Co., Ltd. (Shanghai, China). Allyl bromide and potassium carbonate (analytical grade) were obtained from Shanghai Aladdin Biochemical Technology Co., Ltd. (Shanghai, China) Acetone, dichloromethane, and acetonitrile were of analytical grade and were used as received.

### 2.2. Synthesis of Tri-Allyl Derivative of Curcumin (AEC)

AEC was prepared via Williamson etherification and C-alkylation. Curcumin was reacted with a large excess of allyl bromide in refluxing acetone in the presence of potassium carbonate for 24 h [26]. The crude product (yellow oil, 89% recovery) was purified via silica-gel column chromatography (eluent: dichloromethane/acetonitrile). The target (AEC) was isolated in 80% yield. The structure of AEC was confirmed by NMR, IR, and mass spectrometry (see the Results and Discussion section).

### 2.3. Preparation of BDM-Based Prepolymers and Cured Resins

The details of the preparation of the prepolymers are shown in Table 1. In a typical batch, calculated amounts of BDM and either DABPA or AEC were loaded into a reactor. The mixture was stirred at 150 °C until a homogeneous, clear liquid prepolymer formed. The DABPA-containing prepolymers were denoted BD-*n* and the AEC-containing ones BA-*n*, where *n* is the molar ratio of imide:allyl groups. Each prepolymer was transferred into a preheated mold and vacuum-degassed at 150 °C for 30 min. The resin was then cured using a stepwise thermal schedule: 150 °C for 2 h, 180 °C for 2 h, 200 °C for 2 h, 220 °C for 2 h, and 240 °C for 4 h. After curing, the mold was cooled to room temperature. The cured samples were labeled BDM/DABPA-*n* (BD-*n*) or BDM/AEC-*n* (BA-*n*), according to the formulations, using the same numbering *n* as the prepolymers. A control BDM/AEC sample was cured under the same protocol.

## 3. Results and Discussion

### 3.1. Synthesis and Characterization of AEC

**Figure 2:**
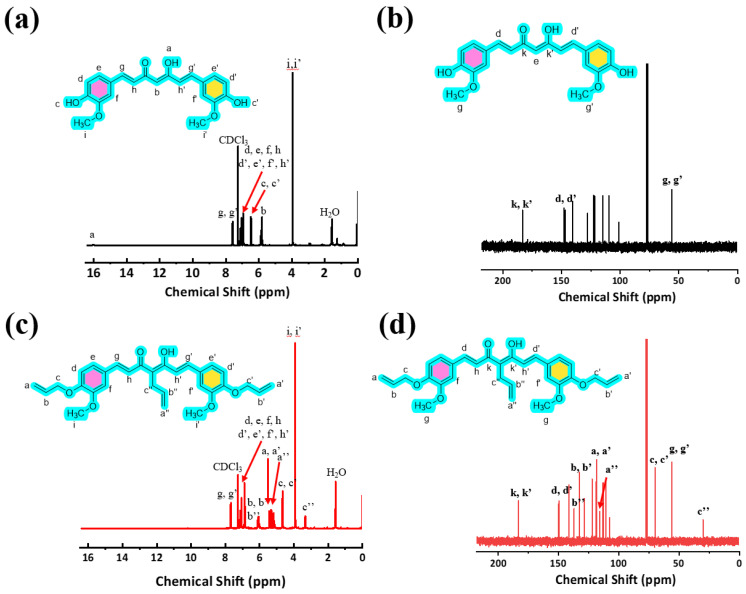
Structural characterization of AEC: (**a**,**b**) ^1^H NMR and ^13^C NMR of curcumin; (**c**,**d**) ^1^H NMR and ^13^C NMR of AEC.

Curcumin was successfully converted to its (AEC) via Williamson etherification and C-alkylation, affording tri-allyl curcumin in 80% yield [26]. Its structure was confirmed by complementary spectroscopy. The ^1^H NMR and ^13^C NMR spectrum of AEC (Figure 2c,d) shows the disappearance of the phenolic –OH signals (δ ~6.4 ppm) and appearance of allyl –CH=CH_2_ signals (δ ~3–5 ppm), consistent with complete etherification (Figure 2a,b). The FT-IR spectrum (Figure S1) of AEC lacks the broad O–H stretch of curcumin and displays new C–O–C bands at ~1250 cm^−1^. High-resolution MS (Figure S2) shows a molecular ion at *m/z* matching (C_30_H_32_O_6_ + H) ^+^ calcd 489.2272 [M]^+^, found: 489.2249 [M]^+^. Together, these data confirm the AEC structure. Curcumin exists in enol and keto forms; the NMR pattern indicates that AEC retains such a structure.

### 3.2. Curing Behavior of BD/BA Resin

**Figure 3:**
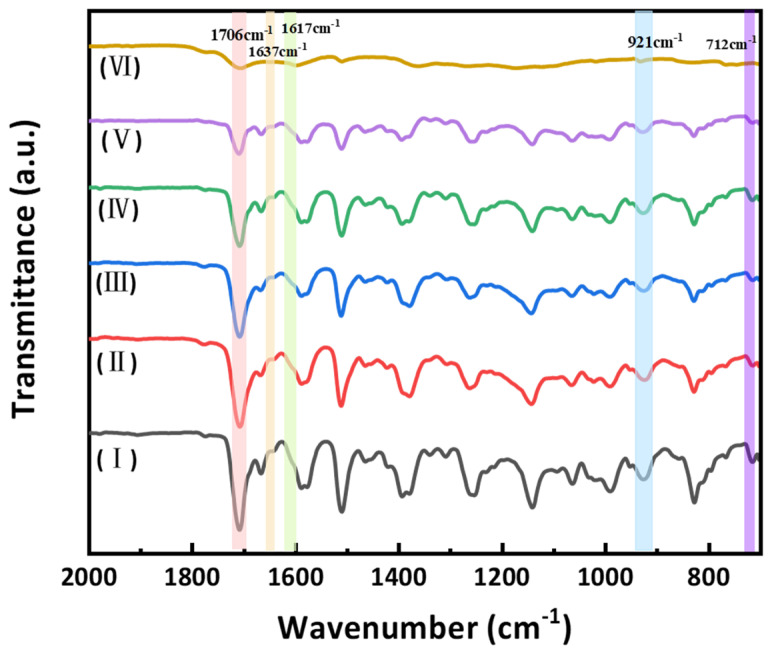
FT-IR spectra of BA − 0.87 during staged curing I–VI (I: Prepolymer; II: +150 °C/2 h; III: +180 °C/2 h; IV: +200 °C/2 h; V: +220 °C/2 h; VI: +240 °C/4 h), as defined above. Key band assignments: allyl C=C (1637 cm^−1^), =C–H (921 cm^−1^), maleimide C=O (1617 cm^−1^), and ring deformation (712 cm^−1^).

**Figure 4:**
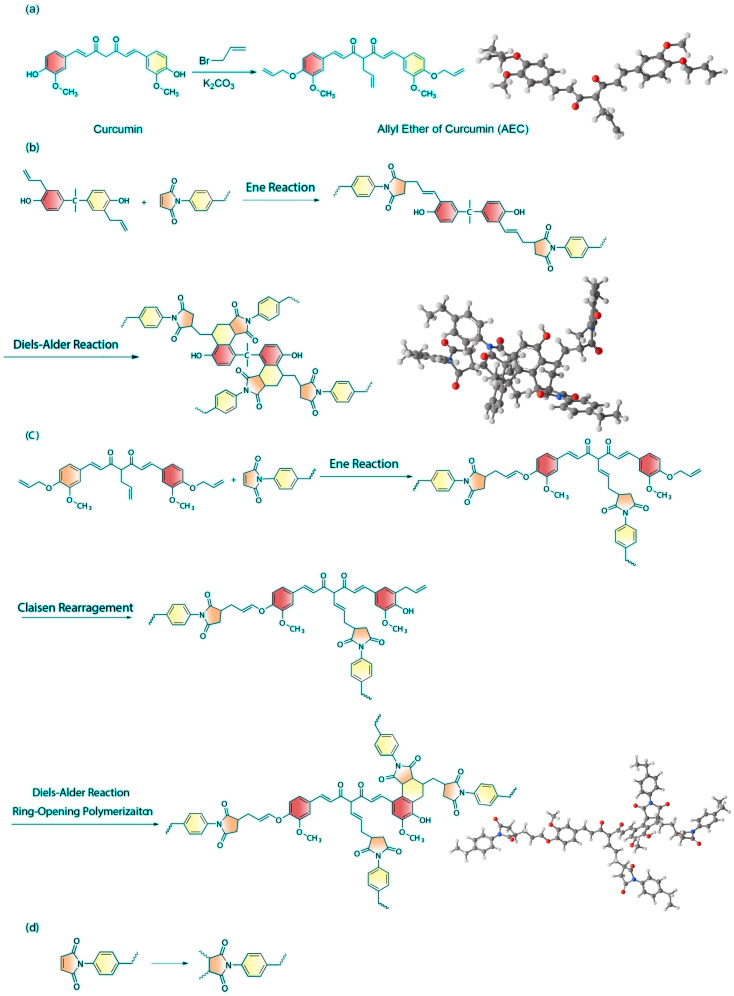
Copolymerization of BD (allyl and imide groups) resins. (**a**) Chemical reaction of AEC, (**b**) BD (DABPA and imide groups) resins, (**c**) and BA (AEC and imide groups) resins, and (**d**) self-polymerization of imide groups.

We monitored the sequential curing of BA-0.87 by FT-IR (Figure 3). Samples were cured in stages (I: Prepolymer; II: +150 °C/2 h; III: +180 °C/2 h; IV: +200 °C/2 h; V: +220 °C/2 h; VI: +240 °C/4 h). In Stages I–IV (≤200 °C), the allyl C=C stretch at 1637 cm^−1^ and out-of-plane =CH wag at 921 cm^−1^ steadily decreased, along with the maleimide C=O asymmetric stretch at 1617 cm^−1^ and ring deformation at 712 cm^−1^. This indicates dominant Michael “ene” addition between AEC’s allyl groups and BDM’s maleimide groups. After Stage IV, residual allyl peaks remain weak. Upon heating to Stage IV (>200 °C), the 921 cm^−1^ allyl band further declines, suggesting that sterically hindered allyl ethers undergo Claisen rearrangement to allylphenols, which then add to maleimides or undergo Diels–Alder cycloaddition. Simultaneously, the 1617 cm^−1^ peak continues to diminish, consistent with the self-polymerization of maleimide double-bonds at high temperatures. The cure mechanism thus involves three integrated pathways: (i) allyl–maleimide copolymerization (dominant < 200 °C), (ii) Claisen rearrangement-assisted crosslinking, and (iii) maleimide self-addition (>200 °C) (schematic in Figure 4b–d).

### 3.3. Curing Reactivity

**Figure 5:**
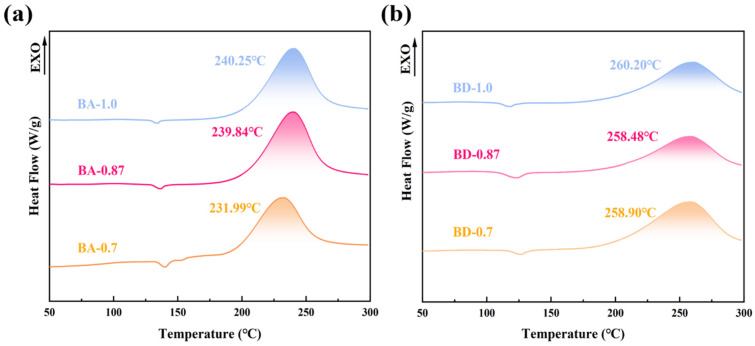
DSC thermograms (10 °C/min, N_2_) for (**a**) BDM/AEC (BA) prepolymers and (**b**) BDM/DABPA (BD) prepolymers. BA curves show lower peak temperatures than BD.

The curing behavior of BD and BA resins was characterized by DSC. Each resin exhibited a single exothermic peak, indicating a dominant curing reaction. The peak curing temperature of BD resin was approximately 260 °C, while BA resin showed a lower peak temperature at 240 °C (Figure 5a). Reducing the peak heat release during curing prevents the excessive heat generation caused by rapid crosslinking. A lower heat release profile enables better temperature control during resin curing, minimizing thermal stress accumulation and thermal runaway risks in thick-section composites (Figure 5b). This significantly broadens the processing window for large composite structural components [27]. Lower curing exothermic peaks effectively prevent thermal runaway in thick-section composite materials, resulting in a more uniform temperature distribution during curing. This is critical for forming large aerospace structural components, as high exothermic peaks can cause excessive internal temperature gradients, leading to voids and internal stresses. The exothermic peak of thermosetting polyimide resins (e.g., PMR-15) in DSC analysis is primarily observed within the temperature range of 275–325 °C [28,29]. Conversely, low exothermic peaks ensure superior dimensional stability and lower defect rates [30]. Three potential factors may contribute to this temperature shift: (i) Claisen rearrangement within the allyl ether of curcumin (AEC) structure could lower activation energy; (ii) increased crosslinking density from AEC’s higher functionality may restrict molecular chain diffusion, thereby distributing heat release over a broader temperature range rather than concentrating it at a single peak; and (iii) concurrent reactions between BDM and multiple AEC sites may spatially disperse exothermic events. Distinguishing between these mechanisms requires further kinetic analysis beyond the scope of this thermal screening.

**Figure 6:**
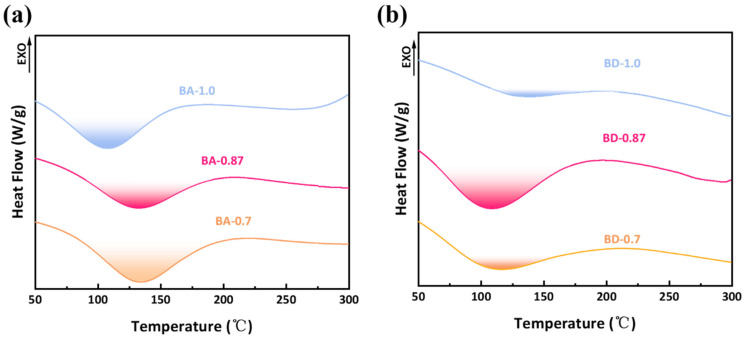
DSC of (**a**) BA and (**b**) BD fully cured resins; cure peaks are eliminated in cured samples.

Curing completeness was verified by comparing the DSC thermograms of prepolymers and fully cured resins. Prepolymer samples for BDM/AEC-0.7, BDM/AEC-0.87, and BDM/AEC-1.0 compositions displayed exothermic peaks, whereas post-cure samples showed no residual exothermic activity (Figure 6a), confirming complete reaction under the applied curing protocol. Post-cure DSC scans show no residual exotherms for any fully cured sample (BA-0.7, BA-0.87, BA-1.0), confirming complete cure under the applied protocol (Figure 6b). Prepolymer scans (Figure 5) all exhibit a single-cure exotherm, which disappears after the staged cure, as expected.

### 3.4. Thermal Stability of Cured Resins

**Figure 7:**
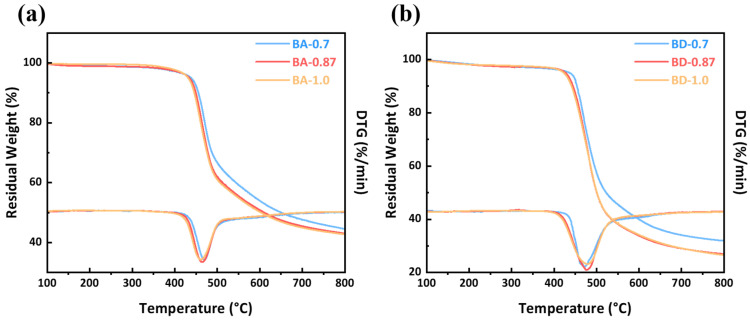
TGA and DTG curves (N_2_, 20 °C/min) for (**a**) BA and (**b**) BD cured resins. BA resins show higher residual mass.

Thermal decomposition under nitrogen was evaluated by TGA, as shown in Figure 7 and Table 2. The onset of decomposition (T_d5%_) ranged from ~423 °C to 438 °C; BA-0.7 showed the highest T_d5%_ (~438 °C). The maximum-rate temperatures (T_dmax_ from DTG) were between 462 and 478 °C for all samples. Char yield at 800 °C (Y_800_) differed markedly: BA-0.87 retained 43.06% mass, whereas BD-0.87 retained only 26.95%, an absolute increase of 16.11 percentage points (relative increase of 59.8%). The enhanced char formation of BA resins is attributed to curcumin’s conjugated β-diketone structure, which promotes carbonization. Among the resin samples, increasing the allyl content (0.7 → 0.87 → 1.0) led to slightly lower char yields: BA resins dropped from 44.58% (BA-0.7) to 42.77% (BA-1.0) (an absolute decrease of 1.81 percentage points), while BD resins fell from 31.97% to 26.53% (an absolute decrease of 5.44 percentage points). Using Van Krevelen’s relation (LOI = 17.5 + 0.4 × Y_800_), the predicted LOI is ~34.7% for BA-0.87 versus 28.3% for BD-0.87. (Experimentally, BA-0.87 achieved LOI ≈ 37.4%, consistent with the higher char yield). Empirical correlation models using elemental ratios (e.g., Van Krevelen diagrams) are convenient for biomass characterization, but suffer from the “Mixture Principle Fallacy.” They oversimplify heterogeneous biomass by reducing it to atomic ratios, ignoring distinct molecular structures and differential thermodynamic contributions (e.g., aromatic vs. aliphatic compounds). These models cannot distinguish structural isomers with identical compositions but different combustion enthalpies and reaction kinetics due to varying bond dissociation energies. Thus, linear regressions based solely on elemental analysis inadequately capture microscopic characteristics, limiting their accuracy for precise thermodynamic modeling [31,32].

### 3.5. Thermomechanical Properties (Tg and Storage Modulus)

**Figure 8:**
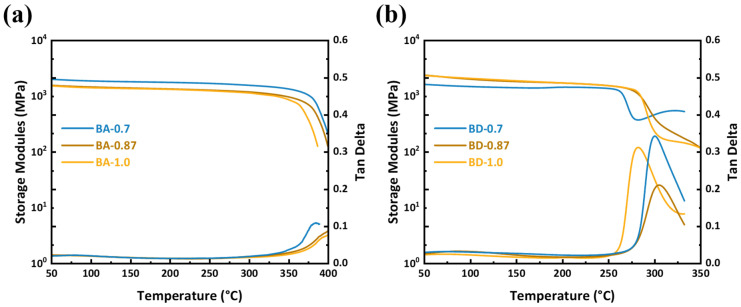
DMA (tan δ) of cured resins: (**a**) BDM/AEC (BA) series; (**b**) BDM/DABPA (BD) series.

All BA resins have much higher *T*_g_ than their BD counterparts. BA-0.7 and BA-0.87 exceed the DMA’s upper limit (>400 °C), and BA-1.0 shows *T*_g_ = 382.5 °C (Figure 8). In contrast, BD-0.7, BD-0.87, and BD-1.0 have *T*_g_ = 305.1, 300.8, and 282.7 °C, respectively (Figure 8b). Thus, even the most flexible BA (BA-1.0) is ~77 °C higher in *T*_g_ than the stiffest BD (BD-0.7). In both series, increasing allyl (ether) content lowers *T*_g_, consistent with lower crosslink density. The uniformly high *T*_g_ of BA resins indicate excellent thermal resistance, suitable for extreme service temperatures. However, the storage modulus of BA-0.87 at 50 °C is recorded as 1561 MPa, which is considerably lower than that of BD-0.87 (2341 MPa). The AEC molecular backbone exhibits high rigidity, trifunctionality, and large volume. Following the curing process, a substantial increase in crosslinking density is observed, resulting in an elevated *T*_g_. However, the large steric hindrance of the latter also results in a more heterogeneous network topology. The bifunctional flexible DABPA molecules have been shown to form a more uniform network structure with fewer segmental defects. It can thus be concluded that, despite the increased crosslinking density driving the increase in *T*_g_, network heterogeneity and local defects reduce load transfer efficiency, leading to a decrease in the overall modulus. While the comparison involves different network topologies, this is a desired outcome of the molecular design: using a bio-based trifunctional node to overcome the thermal limitations of linear spacers. Furthermore, at elevated temperatures, AEC may undergo Claisen rearrangement and Diels–Alder side reactions. It has been demonstrated that these reactions increase crosslinking and further elevate *T*_g_. In addition, they enhance local stiffness and stress concentration, thereby suppressing the growth of the material’s energy storage modulus [33,34].

### 3.6. Mechanical Properties

**Figure 9:**
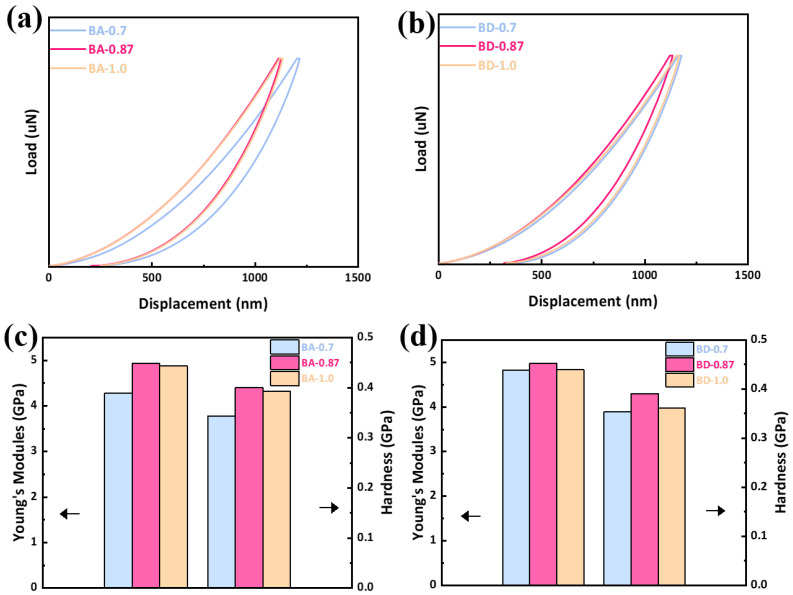
Nanoindentation results (10 mN load) for (**a**) BA-cured resins of load-displacement curves (**b**) BD-cured resins of load-displacement curves. (**c**) BA-cured resins of Young’s modules and hardness. (**d**) BD-cured resins of Young’s modules and hardness. Higher modulus and hardness indicates a stiffer network.

Nanoindentation (Figure 9, Table 3) shows composition-dependent stiffness. In the BA series, BA-0.87 has the highest Young’s modulus (*E* = 4.94 GPa) and hardness (*H* = 0.44 GPa), with the shallowest maximum penetration (h_max_ = 1129 nm). These values represent an increase of 0.67 GPa (15.7%) for E and 0.06 GPa (15.8%) for H compared to BA-0.7 (4.27 GPa, 0.38 GPa), and are slightly above BA-1.0 (4.88 GPa, 0.43 GPa). In the BD series, BD-0.87 peaks at *E* = 4.97 GPa, *H* = 0.43 GPa. The optimal stiffness at intermediate AEC content (0.87) suggests a balanced network with minimal residual stress. While nanoindentation confirms high modulus and local hardness, macro-scale toughness (e.g., impact strength) was not evaluated in this study. Future work will address the impact of the rigid AEC network on fracture mechanics.

### 3.7. Flame Retardancy Performance

**Figure 10:**
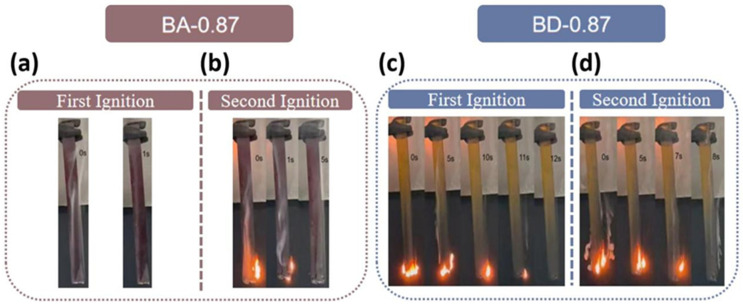
UL-94 vertical burn tests: first (**a**,**c**) and second (**b**,**d**) ignitions for (**a**,**b**) BD-0.87 and (**c**,**d**) BA-0.87. BA-0.87 quenches more rapidly and produces no melt-drip.

**Figure 11:**
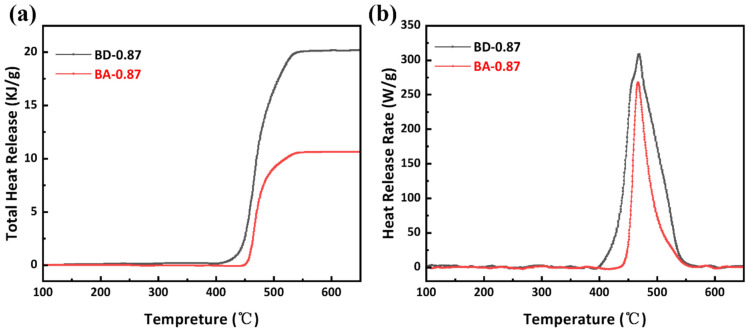
(**a**) MCC total heat release (THR); (**b**) heat release rate (HRR) for cured BA-0.87 (red) and BD-0.87 (black) resins.

The flame tests (LOI, UL-94) and MCC results are summarized in Table 4 and Figure 10. BA-0.87 achieves LOI = 37.4%, an absolute increase of 9.9 percentage points compared to BD-0.87 (30.5%), reflecting its higher char yield. In UL-94, BA-0.87 reaches V-0 (burn times < 10 s, no dripping), whereas BD-0.87 only achieves V-1 (<30 s, no drip). Microscale combustion calorimetry (Figure 11) quantifies heat release: BA-0.87 has PHRR = 267.9 W/g, 40.8 W/g lower than BD-0.87 (308.7 W/g). Total heat release (THR) is 10.7 kJ/g for BA-0.87 vs. 20.2 kJ/g for BD-0.87 (an absolute decrease of 9.5 kJ/g). The heat release capacity (HRC) also decreases from 305.6 J/g·K (BD-0.87) to 265.3 J/g·K (BA-0.87), indicating substantially reduced flammability.

In UL-94 testing, BA-0.87 achieved a V-0 rating with burning times < 10 s for both ignitions and no dripping observed (Figure 10a,b). In contrast, BD-0.87 achieved V-1 classification with burning times < 30 s and no dripping (Figure 10c,d).

MCC analysis quantified the heat-release parameters under controlled pyrolysis conditions (Table 4). BA-0.87 exhibited a peak heat release rate (PHRR) of 267.9 W/g, which was 13.2% lower than BD-0.87 (308.7 W/g). Total heat release (THR) for BA-0.87 (10.7 kJ/g) was approximately half that of BD-0.87 (20.2 kJ/g), representing a reduction of 9.5 kJ/g (47.0%). The heat release capacity (HRC), a predictive indicator of material flammability, was lower for BA-0.87 (265.3 J/g·K) compared to BD-0.87 (305.6 J/g·K), indicating a reduced flammability potential 40.3 J/g·K.

### 3.8. Flame-Retarding Mechanism

**Figure 12:**
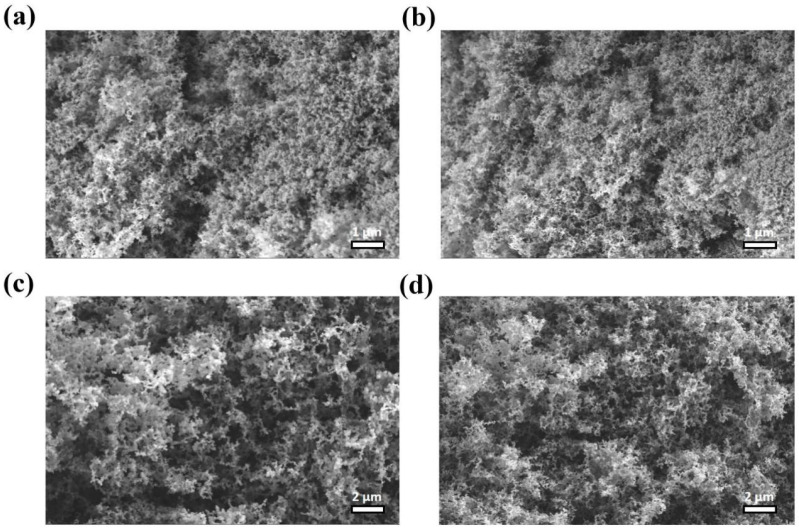
SEM images of residual chars from UL-94 tests: (**a**,**b**) BD-0.87, (**c**,**d**) BA-0.87. Both show porous carbon networks.

SEM imaging of residual char surfaces after LOI testing revealed similar porous microstructures for both BD-0.87 and BA-0.87 (Figure 12a–d), with interconnected pore networks permeable to gas molecules. The morphological similarity suggested that differences in flame retardancy were not primarily attributable to the effects of the physical barriers from the density of the char layer.

**Figure 13:**
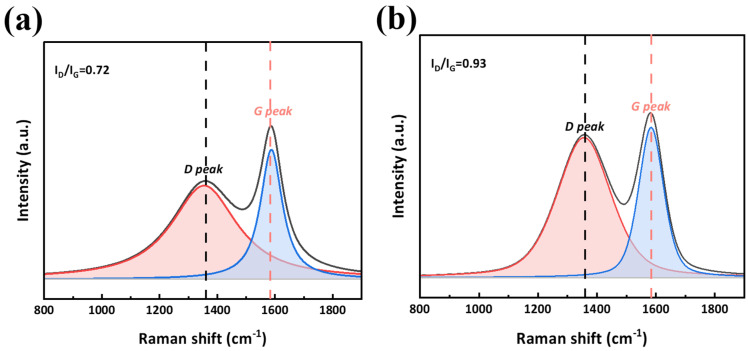
Raman spectra of chars from (**a**) BA-0.87; (**b**) BD-0.87 resins. The defect-related D peak (red bars) and the graphitic G peak (blue bars).

Raman spectroscopy analysis of residual chars allowed for the quantitative assessment of graphitization degree through the intensity ratio of D-peak (~1350 cm^−1^, defect/disorder) to G-peak (~1580 cm^−1^, ordered *sp*^2^ carbon) (Figure 13). BA-0.87 char has lower I_D_ < I_G_ (0.72) than BD-0.87 (0.93), indicating higher graphitization. BA-0.87 char exhibited I_D_/I_G_ = 0.72, indicating substantial graphitization with predominantly *sp*^2^-hybridized carbon domains and low defect density. BD-0.87 char showed I_D_/I_G_ = 0.93, representing a 0.21 increase and indicating lower graphitization with more disordered regions or structural defects.

**Figure 14:**
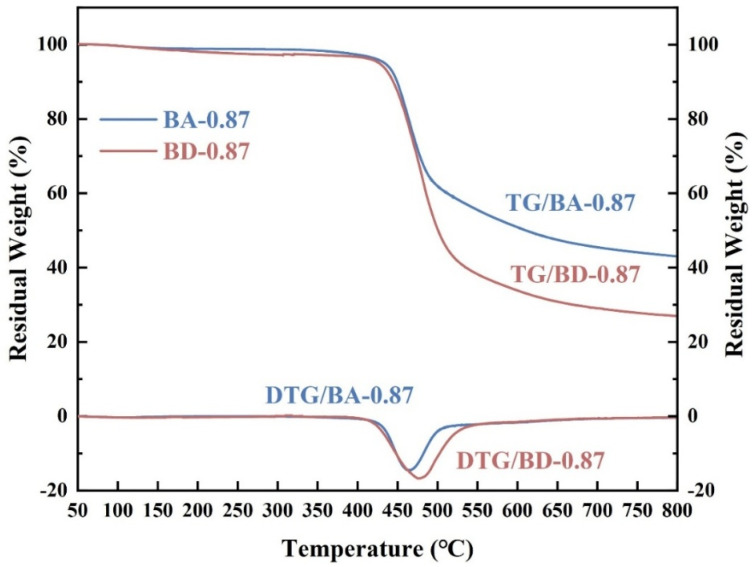
TG and DTG curves (N_2_) for BA-0.87 (blue) and BD-0.87 (red) from TG-IR analysis, confirming higher residual char for BA-0.87.

TG-IR analysis (Figure 14) further elucidates degradation pathways. BA-0.87 retained 43.06% char at 800 °C vs. 26.95% for BD-0.87, confirming enhanced carbonization. The DTG peak of BA-0.87 is lower, indicating slower mass loss.

**Figure 15:**
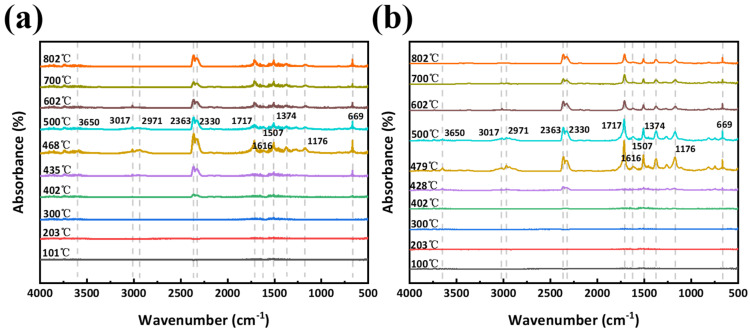
FT-IR spectra of pyrolysis gases from (**a**) BA-0.87 and (**b**) BD-0.87 at key decomposition temperatures.

**Figure 16:**
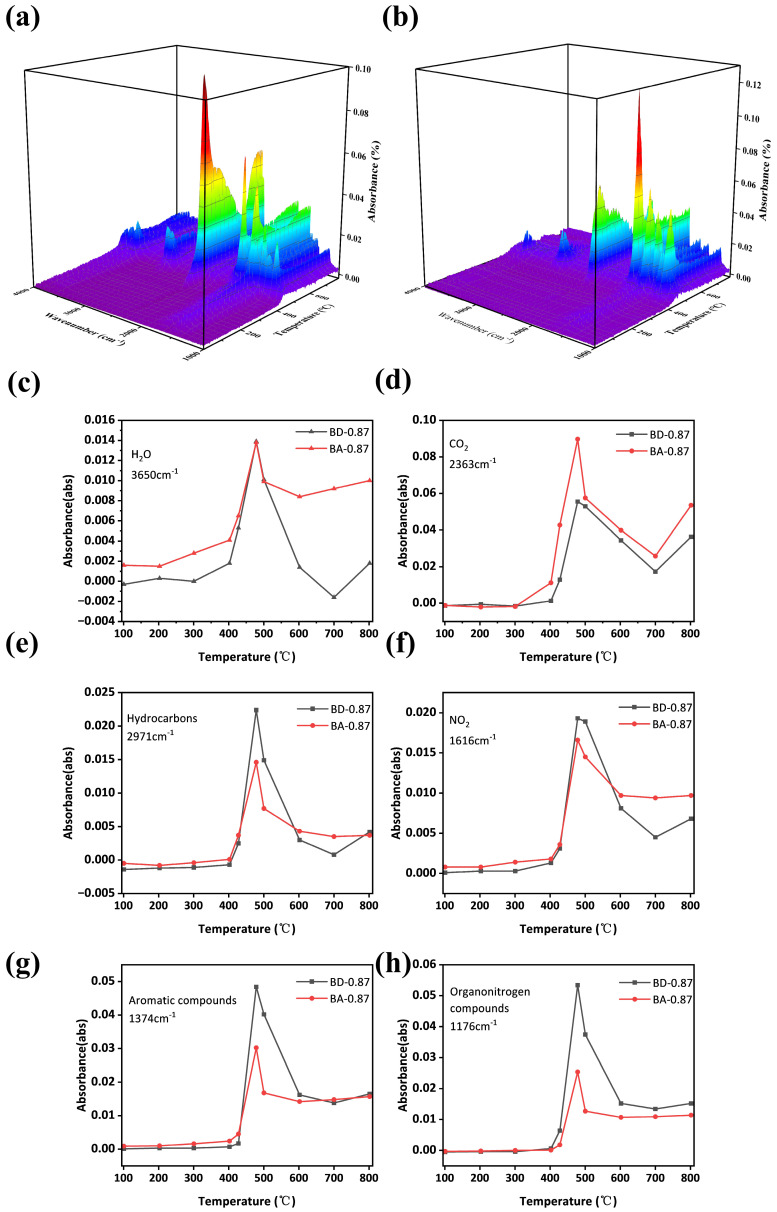
Three-dimensional FT-IR plots of evolved gases (absorptivity vs. wavenumber vs temperature) for (**a**) BA-0.87 and (**b**) BD-0.87; concentration profiles of main pyrolysis products vs. temperature: (**c**) H_2_O, (**d**) CO_2_, (**e**) hydrocarbons, (**f**) NO_2_, (**g**) aromatic compounds, (**h**) Organonitrogen for BA-0.87 (red) and BD-0.87 (black). BA-0.87 emits more H_2_O and CO_2_ (**c**,**d**) and less combustibles (**e**,**g**,**h**).

FT-IR of evolved gases (Figure 15 and Figure 16) shows that BD-0.87 emits more flammable volatiles: hydrocarbon (3017, 2971 cm^−1^), NO_2_, and carbonyl (1717 cm^−1^) peaks appear at 428–479 °C. In contrast, BA-0.87 releases fewer combustible species. Quantitative absorbance vs. temperature profiles (Figure 16) demonstrate that BA-0.87 emits relatively more non-combustibles (H_2_O, CO_2_) and less hydrocarbons, aromatics, and nitrogenous organics. In summary, curcumin’s structure promotes char formation and favors nonflammable degradation products, thereby lowering heat release and enhancing flame retardancy.

## 4. Conclusions

We developed bismaleimide (BDM) thermosets modified with a curcumin-derived allyl ether (AEC) co-monomer. Compared to the conventional DABPA-based benchmark, AEC-containing resins cure at lower temperatures. Notably, the BA-0.87 formulation (*imide:allyl* = 0.87) exhibits the best overall performance: *T*g > 400 °C, decomposition onset (*T*_d5%_) ≈ 434 °C, and very high char yield (~43% at 800 °C). Crucially, BA-0.87 passes UL-94 V-0 without dripping. These gains stem from curcumin’s rigid, conjugated backbone, which increases crosslink density and char formation during combustion. In summary, AEC serves as a drop-in bio-based replacement for DABPA that simultaneously improves thermal stability and intrinsic flame retardancy. This work thus introduces a new bio-derived BMI resin suitable for demanding high-temperature and fire-resistant applications while eliminating the health concerns associated with bisphenol-based modifiers.

## Figures and Tables

**Table 1:** Formula of different resins.

Resin	BDM (g)	DABPA (g)	AEC (g)	BDM (mol)	DABPA (mol)	AEC (mol)
BD-0.7	5	3.08	-	0.014	0.010	-
BD-0.87	5	3.7	-	0.014	0.012	-
BD-1.0	5	4.3	-	0.014	0.014	-
BA-0.7	5	-	2.93	0.014	-	0.006
BA-0.87	5	-	3.91	0.014	-	0.008
BA-1.0	5	-	4.40	0.014	-	0.009

**Table 2:** Thermal and thermomechanical properties of cured resins (from TGA and DMA).

Resin Code	T_d5%_ (°C)	T_dmax_ (°C)	Y_800°C_	T_g_ (°C)	Storge Modulus (MPa)
BA-0.7	438.03	469.19	44.58%	>400	2004
BA-0.87	433.96	464.64	43.06%	>400	1561
BA-1.0	431.12	461.82	42.77%	382.53	1527
BD-0.7	436.96	472.00	31.97%	305.08	2380
BD-0.87	427.07	475.93	26.95%	300.79	2341
BD-1.0	423.04	478.28	26.53%	282.73	1624

**Table 3:** Nanoindentation properties of cured resins (five indents per sample).

Resin Code	Young’s Modulus *E* (GPa)	Hardness *H* (GPa)	h_(Max) (nm)
BA-0.7	4.27	0.38	1219
BA-0.87	4.94	0.44	1129
BA-1.0	4.88	0.43	1136
BD-0.7	4.82	0.39	1180
BD-0.87	4.97	0.43	1138
BD-1.0	4.84	0.40	1171

**Table 4:** Flame-retardancy test results for BA-0.87 vs. BD-0.87.

Resin Code	LOI %	UL-94 Level	PHRR (W/g)	THR (kJ/g)	HR Capacity (J/g·K)	Temperature (°C)
BA-0.87	37.4	V-0	267.9	10.7	265.3	468.6
BD-0.87	30.5	V-1	308.7	20.2	305.6	468.2

## Data Availability

The raw data supporting the conclusions of this article will be made available by the authors on request.

## Supplementary Materials

The following supporting information can be downloaded at https://www.mdpi.com/article/10.3390/polym18030399/s1. Figure S1: FT-IR spectra of curcumin and AEC; Figure S2: HR-ESI MS of AEC; Figure S3: 1H NMR of AEC.
