# Supplementary material for: Curcumin-Based Tri-Allyl Derivative for Bismaleimide Resins: Synergistic Modulation of Thermal Stability and Fire Safety
Source: Polymers (Basel). 2026 Feb 3;18(3):399. doi: 10.3390/polym18030399 (PMC12899180; doi:10.3390/polym18030399)
Supplement: Supplementary file 1 [file polymers-18-00399-s001.zip › polymers-4095528-supplementary.pdf]

Supplementary Information

Curcumin-Based Tri-Allyl Derivative for Bismaleimide Resins: Synergistic Modulation of Thermal Stability and Fire Safety

Hui Liu[a], Teresa Olszewska[b]*, Hao Liu[a]**

[a] State Key Laboratory of Advanced Fibers Materials, Center for Advanced Low-dimension Materials, College of Materials Science and Engineering, Donghua University, Shanghai, 201620, PR China

[b] Department of Organic Chemistry, Faculty of Chemistry, Gdańsk University of Technology, 80-233 Gdańsk, Poland

Keywords: bismaleimide resin; curcumin; bio-based polymer; flame retardancy; char yield

Table of Content

1. Characterization………………………………………………………………….3

2. Supplementary figures……………………………………………………….......9

1 | Characterization

^{1}H NMR and ^{13}C NMR spectra were recorded on a Bruker 400 MHz spectrometer (Germany) using deuterated chloroform ($CDCl_3$) as the solvent and tetramethylsilane (TMS) as an internal reference. Chemical shifts were reported in ppm. High-Resolution Mass Spectrometry (HRMS) was performed on an AB SCIEX 5800 matrix-assisted laser desorption/ionization time-of-flight liquid chromatography/mass spectrometry (MALDI-TOF LC/MS) apparatus (USA). Fourier Transform Infrared (FT-IR) spectra were obtained using a Bruker TENSOR II spectrometer (Germany) equipped with a diamond attenuated total reflection (ATR) stage. Scans were conducted over 500–4000 cm^{-1}. Differential Scanning Calorimetry (DSC) curves were obtained on a TA Instruments Discovery DSC250 (USA) under a nitrogen atmosphere from 30-300℃ (flow rate: 50 mL/min) at a heating rate of 10 °C/min. Dynamic Mechanical Analysis (DMA) was performed on a Mettler DMA Q800 apparatus (USA) in multifrequency strain mode with a film tension clamp. Samples (25 ± 0.2 mm × 5 ± 0.2 mm × 3.0 ± 0.05 mm) were heated from 30 °C to 400 °C at 3 °C/min under a nitrogen atmosphere with a frequency of 1.0 Hz. Thermogravimetric Analysis (TGA) and Differential Thermogravimetry (DTG) were conducted on an TGA4000-FTIR system (PerkinElmer, USA) under a nitrogen atmosphere heating from 30 °C to 800 °C at 20 °C/min (flow rate: 50 mL/min). Micromechanical properties were measured using a nanoindenter instrument (Bruker Hysitron TI 980, Germany) equipped with a Berkovich diamond indenter (probe radius: 30 nm). Indentation force was loaded to 10,000 μN at a rate of 400 μN s^{-1}, held for 2 s, and unloaded at a rate of 400 μN s^{-1}. Number of indentation points per sample: 5. Limiting oxygen index (LOI) was tested per ASTM D2863-17 using a 5801-A apparatus equipped with a magneto-dynamic oxygen analyser. Specimens measured 130 mm × 6.5 mm × 3 mm. Vertical burning (UL-94) tests were conducted according to ASTM D3801-10 on a 5402-A instrument. Specimens measured 130 mm × 13 mm × 3 mm. Microscale Combustion Calorimetry (MCC) was performed on a Federal Aviation Administration (FAA) microcalorimeter (model: DEATAK MCC-3, USA). Heating rate: 1℃/s. Scanning Electron Microscopy (SEM)

images were acquired using a ZEISS GeminiSEM 300 microscope at 3.0KV. Laser confocal Raman spectroscopy was performed using an Thermo Fisher Scientific DXR2xi (USA) with a 532 nm argon-ion laser. Thermogravimetric Analysis-Infrared Spectroscopy (TG-IR) spectra were obtained using a coupled TGA4000-FTIR system (PerkinElmer, USA). Samples (10 ± 0.02 mg) were heated from 30 °C to 800 °C at 20 °C/min under nitrogen (flow rate: 50 mL/min).

2 | Supplementary figures

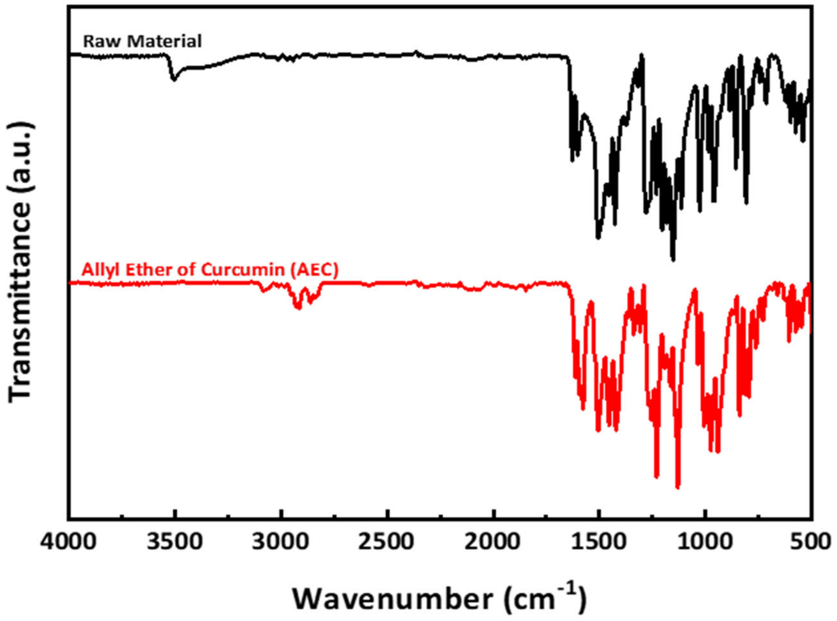

Fig. S1 FT-IR spectra of curcumin and AEC

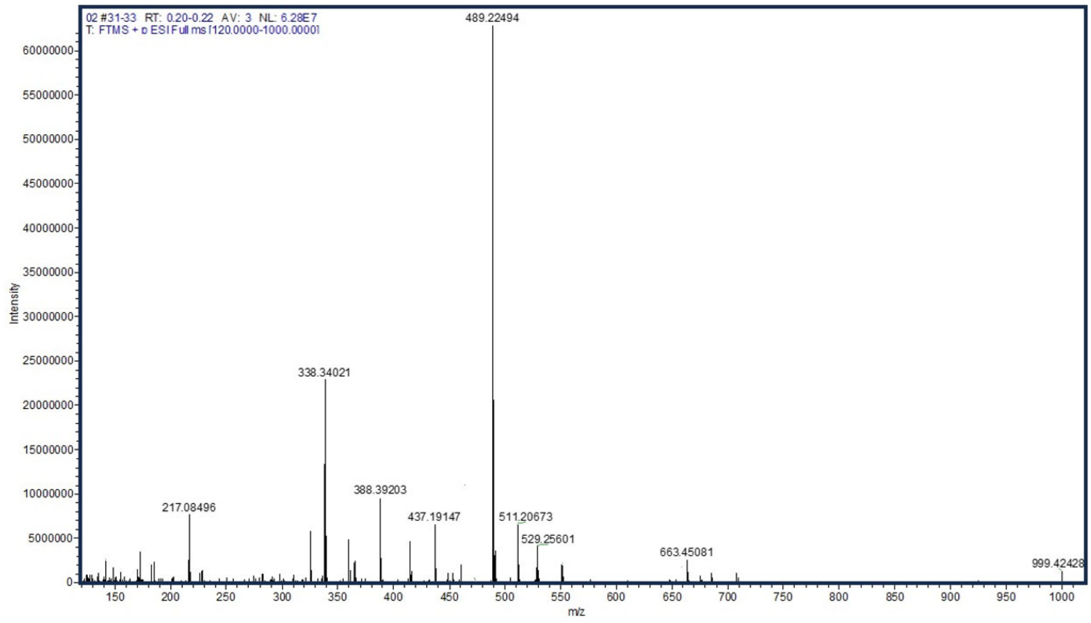

Fig. S2 HR-ESI MS of AEC

^{1}H NMR (400 MHz, $CDCl_3$) δ 7.65 (m, 2H, g,g'), 7.24 (s, $CDCl_3$), 6.97 (m, 8H, d,d',e,e',f,f',h,h'), 6.05 (m, 3H, b,b',b'''), 5.39 (m, 2H, a,a'), 5.11 (m, 1H, a''), 4.64 (m, 2H, c,c'), 3.90 (m, 2H, i,i'), 3.29 (m, 1H, c''), 1.54 (s, H_2O) ppm.

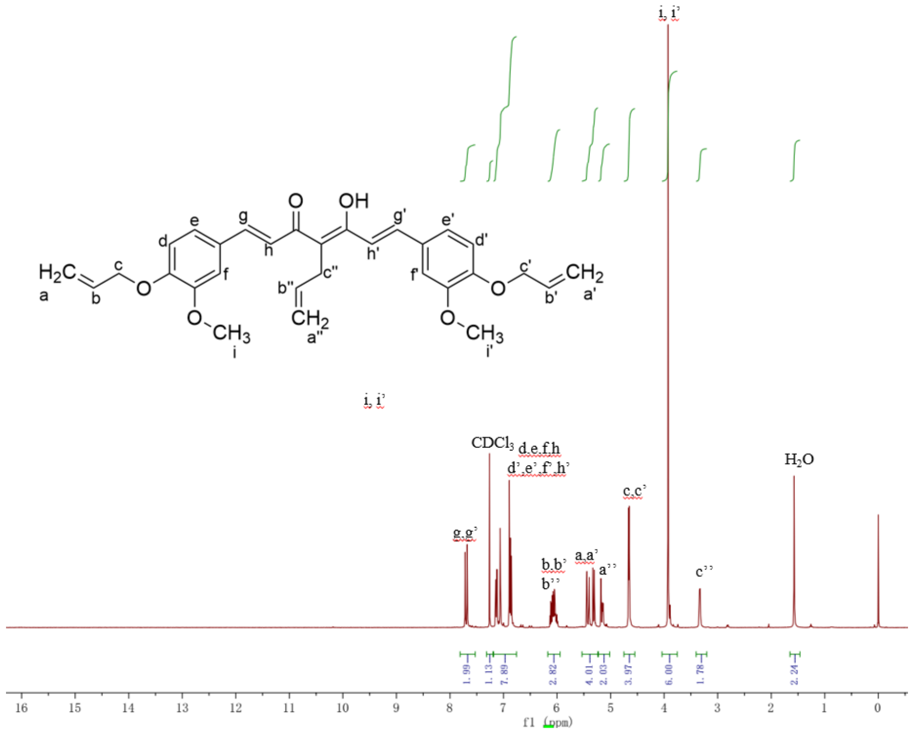

Fig. S3 1H NMR of AEC
